# Validating the weight gain of preterm infants between the reference growth curve of the fetus and the term infant

**DOI:** 10.1186/1471-2431-13-92

**Published:** 2013-06-11

**Authors:** Tanis R Fenton, Roseann Nasser, Misha Eliasziw, Jae H Kim, Denise Bilan, Reg Sauve

**Affiliations:** 1 Department of Community Health Sciences, Faculty of Medicine, Alberta Children’s Hospital Research Institute, The University of Calgary, 3280 Hospital Drive NW, Calgary, AB T2N 4Z6, Canada; 2 Regina Qu’Appelle Health Region, 1440-14th Avenue, Regina, Sask S4P 0W5, Canada; 3 Department of Public Health & Community Medicine, Tufts University - School of Medicine, 136 Harrison Avenue, Room 114, Boston, MA 02111, USA; 4 Division of Neonatology, UC San Diego Medical Center, 200 West Arbor Drive MPF 1140, San Diego, CA 92103-8774, USA; 5 Department of Pediatrics, The University of Calgary, Calgary AB T2N 4Z6, Canada

## Abstract

**Background:**

Current fetal-infant growth references have an obvious growth disjuncture around 40 week gestation overlapping where the fetal and infant growth references are combined. Graphical smoothening of the disjuncture to connect the matching percentile curves has never been validated. This study was designed to compare weight gain patterns of contemporary preterm infants with a fetal-infant growth reference (derived from a meta-analysis) to validate the previous smoothening assumptions and inform the revision of the Fenton chart.

**Methods:**

Growth and descriptive data of preterm infants (23 to 31 weeks) from birth through 10 weeks post term age were collected in three cities in Canada and the USA between 2001 and 2010 (n = 977). Preterm infants were grouped by gestational age into 23–25, 26–28, and 29–31 weeks. Comparisons were made between the weight data of the preterm cohort and the fetal-infant growth reference.

**Results:**

Median weight gain curves of the three preterm gestational age groups were almost identical and remained between the 3rd and the 50th percentiles of the fetal-infant-growth-reference from birth through 10 weeks post term. The growth velocity of the preterm infants decreased in a pattern similar to the decreased velocity of the fetus and term infant estimates, from a high of 17–18 g/kg/day between 31–34 weeks to rates of 4–5 g/kg/day by 50 weeks in each gestational age group. The greatest discrepancy in weight gain velocity between the preterm infants and the fetal estimate was between 37 and 40 weeks; preterm infants grew more rapidly than the fetus. The infants in this study regained their birthweight earlier compared to those in the 1999 National Institute of Child Health and Human Development report.

**Conclusion:**

The weight gain velocity of preterm infants through the period of growth data disjuncture between 37 and 50 weeks gestation is consistent with and thus validates the smoothening assumptions made between preterm and post-term growth references.

## Background

Nutrition experts continue to recommend that preterm infants should grow and accrete nutrients at the same rate as the healthy unborn fetus [1-3] and that their growth should be similar to the healthy term infant after 40 weeks [1,3]. The growth rates of the fetus and preterm infant differ and change dramatically with post-menstrual age. From 24 weeks to term, fetuses grow rapidly, multiplying their weight 5 times in a period less than 4 months [4-8]. In comparison, term infants double their birthweight by 4 to 5 months [9]. Noticeably, at the time of discharge from the neonatal intensive care unit (NICU), the weights of most preterm infants are lower than fetal norms [10-16] as preterm infants frequently do not achieve the targeted fetal or early post-term growth rates.

Fetal-infant growth charts are commonly used to track the trajectory of infants. Because fetal-infant growth charts have incorporated two disparate data sets based on the fetus and the term infant, this creates an obvious disjuncture between the two reference data sets. The Babson & Benda growth chart [17], for example, did not describe the smoothing steps of this disjuncture to link their fetal and term infant data sets. The 2003 Fenton growth chart joined the fetal and infant growth reference data by smoothing this disjuncture around 40 weeks gestational age by using computer-assisted graphical methods [18]. Despite the approximations used in the current growth curves, it remains unclear how preterm infants truly grow through the disjuncture period.

The primary objective of this cohort study was to compare weight gain of preterm infants to a meta-analysis estimate of fetal and infant growth [19], specifically focusing on the fetal-infant growth reference disjuncture between 37 to 50 weeks. This cohort’s growth was also compared to a well-cited description of preterm infant growth of infants born in 1994–5 [10]. The study findings then were used to validate and inform the revision of the Fenton growth chart.

## Methods

We compared the postnatal weight gain of preterm infants in three North American cities to a fetal-infant growth reference (FIGR), which was generated based on a systematic review of the literature before 40 weeks [19] and the World Health Organization Growth Standard (WHOGS) after 40 weeks [20] of gestation. The FIGR fetal weight values were a weighted average of fetal growth from six population-based surveys with a minimum required sample size > 25,000 infants from developed countries (Germany, Italy, USA, Canada, Australia, and Scotland) over the past 25 years [4-8,21].

### The preterm infant multicentre growth study (PreMGS)

Growth, medical, nutrition, and descriptive data of preterm infants were collected in three cities: Calgary and Regina in Canada, and San Diego in USA from a retrospective chart review. Neonatal intensive care was provided by Alberta Health Services in Calgary, Regina Qu’Appelle Health Region (RQHR) in Regina, and the NICU at the University of California San Diego (UCSD) Medical Center in San Diego, USA. These neonatal units have provided early nutrition support since 2005. Parenteral nutrition started on the birth day or day one of life for most of these infants. Post-discharge, the infants were cared for in the Neonatal Transition Program and the Southern Alberta Perinatal Clinic in Calgary, the Neonatal Intensive Care Follow-up Program in Regina, and the Premature Infant Nutrition Community (PINC) Clinic in San Diego. The PreMGS was granted ethical approval by the Conjoint Health Research Ethics Board, University of Calgary and RQHR Research Ethics Board Regina, and UCSD Human Research Protections Program.

### Subjects

The preterm infants were included if they were born between 2001 and 2010, and their gestational age was less than 32 weeks. Infants were excluded if they had congenital anomalies or did not survive until discharge, as these conditions could affect growth. The data were collected prospectively during clinical care in Level III NICUs, associated Level II units and in routine post-discharge care, and extracted from the charts by trained research assistants.

### Clinical data

Neonatal information included: gender, gestational age (weeks), medical history of necrotizing enterocolitis, oxygen therapy, SNAP II scores, and anthropometric measurements: a) size at birth (weight (kg), head circumference (cm), and recumbent length (cm)), b) daily weights for the first 21 days, c) weekly size measures (weight, head circumference, and length) while in the hospital, d) all available size measures post discharge up to and including 4 months corrected age. Gestational age was defined by maternal dates and/or ultrasound. If maternal dates differed by more than 2 weeks from assessed age, and if early ultrasound data was not available, then assessed age was used. Appropriateness of size for gestational age was assigned based on the FIGR.

### Nutrition data

Data on nutrition support included: age at initiation of parenteral and enteral nutrition (minimal enteral feedings (less than or equal to 20 mL/kg/day) as well as enteral feedings (greater than 20 mL/kg/day), full enteral feedings (defined as greater than 140 mL/kg/day), weekly recordings of types of feeding in hospital and post discharge (breast milk or formula), use of human milk fortifier (powder or preterm formula as a fortifier), total fluid intakes, and number of feeding interruptions (defined as being stop of enteral/oral feeds with advancement of feeds that took 4 + days to achieve greater than140 mL/kg).

### Data management

The preterm infants were grouped into three cohorts according to their gestational age at birth: 23 to 25, 26 to 28, and 29 to 31 weeks. As not all infants were measured each week after discharge, values were interpolated between measurements. The individual and median weight gain trajectories of the preterm infants were plotted together with the FIGR.

The cohorts’ weights at various ages were compared to the FIGR using z-scores. Specifically we determined the proportion of growth restriction (defined as weight less than the 10th percentile) separated either as intrauterine growth restriction (IUGR) at birth or extrauterine growth restriction (EUGR) any time postnatally. Comparisons were made against the FIGR (for data up to 40 weeks) and the WHOGS (for data 40 to 50 weeks).

Weight gain velocity was calculated (in g/kg/day) along the median weight curves, for the preterm infant cohorts and the FIGR using the follow calculation [22]:

$$\begin{array}{c} \underset{\_}{\text{end weight - start weight in grams}}\\ \underset{\_}{\text{average weight in kilograms}}\\ \text{number of days} \end{array}$$

The smoothed weight gain velocity of the three preterm infant cohorts, together with the median FIGR weight gain velocity, were plotted against gestational age, and weight gain during the intervals were reported. Differences between median FIGR weight velocity and the preterm infants’ weight gain (g/kg/day) were calculated by subtracting the fetal-infant rates from the preterm rates.

The mean weight gain for the three groups of preterm infants in this study were compared to the mean growth of the infants from the National Institute of Child Health and Human Development Neonatal Research Network (NICHD) [10] (born between 1994 and 1995), along with the FIGR-WHOGS 3rd, 50th and 97th percentiles. To obtain an NICHD cohort similar to our 23–25 week cohort (mean birthweight 664 grams) we combined the 550 and 750 gram NICHD cohorts using a weighted average and pooled standard deviations.

### Statistical analysis

Comparisons of the prevalence of growth restriction at various time points were made using two-sample tests of proportions. t-tests were used to compare mean preterm weight gain velocity rates with the mean of the FIGR estimates. Results with p-values < 0.05 were considered to be statistically significant.

## Results

The preterm infants in the three city cohorts had similar average birth weights and gestational ages (Table 1). These infants were started on early parenteral nutrition and most were fed fortified own mothers’ milk as their majority feedings (Table 2 and 3).

**Table 1:** PreM Growth Study Subject characteristics by city*

	**Total**	**Calgary**	**Regina**	**San Diego**
n	977	851	93	34
Birthweight (grams)	947 (220)	943 (216)	938 (174)	1079 (393)
Gestational age (weeks)	27.0 (1.9)	27.0 (1.9)	26.3 (1.3)	27.7 (2.5)

* mean (SD).

**Table 2:** Neonatal, feeding and discharge characteristics of the PreM Growth Study cohorts gestational age categories*

**Characteristic**	**23 to 25 weeks**	**26 to 28 weeks**	**29 to 31 weeks**
N	227	539	213
Birthweight (grams)	715 (124)	997 (194)	1071 (169)
Gestational age (weeks)	24.4 (0.7)	27.1 (0.8)	29.7 (0.8)
Male sex (%)	51.5	51.8	55.2
Necrotizing enterocolitis** (%)	6.1	6.0	4.4
Supplemental O_2_ (days)	92 +/− 36	56 +/− 35	24 +/− 16
On oxygen at 28 days (%)	97.2	78.5	34.7
On oxygen at 36 weeks (%)	66.2	42.8	18.5
Feeding :			
Parenteral nutrition start (day)	0.4 (0.7)	0.4 (0.6)	0.4 (0.7)
Minimal enteral feed start (day)	7.4 (5.9)	4.4 (3.7)	2.7 (3.1)
Enteral feeds start (day)	16.7 (9.4)	9.8 (6.9)	6.6 (5.3)
Full feeds (day)	34 (15)	22 (10)	17 (9.4)
Predominant EN feedings:			
Fortified breastmilk (%)	77.4	77.9	80.4
Non-fortified breastmilk (%)	1.9	2.6	7.6
Preterm formula (%)	15.7	13.3	8.7
Post D/C breastmilk (%)	50.4	52.3	67.2

* mean (SD).

** stage II or III NEC as defined by modified Bell’s criteria [23].

**Table 3:** Neonatal nutrition practices in the PreM Growth Study three cities at the time of the study

	**Calgary**	**Regina**	**San Diego**
A. Nutrition plans:			
Goals for parenteral nutrition			
Protein (g/kg/day)	3.5 to 4	3.5 to 4	4
Lipid (g/kg/day)	3 to 4	3 to 4	3.5
Type of amino acid	Trophamine 2001 to 2006, Primene 2006 to 2010	Aminosyn PF 2001- May 2005 Primene 2005 to 2010	Trophamine
Type of lipids	Intralipid	Intralipid	Intralipid
Beginning doses:			
Protein (g/kg/day)	1-2 grams	1-2 grams	2 grams
Lipid (g/kg/day)	1 gram on day 1 or 2	0.5-1.0 grams on day 1 or 2	1-2 grams
Rate of increment:			
Protein (g/kg/day)	1 gram	0.5-1.0	1 gram
Lipid (g/kg/day)	1 gram	0.5-1.0	1 gram
B. Actual nutrition data:			
Parenteral nutrition start (day)	0.4 (0.6)	0.7 (1.0)	0 (0)
Minimal enteral feed start (day)	4.2 (4.1)	9.2 (5.2)	4.7 (4.0)
Enteral feeds start (day)	9.8 (7.3)	17.4 (9.2)	12.8 (10.5)
Full feeds (day)	22.2 (10.8)	36.2 (17.7)	29.4 (19.7)
C. Predominant hospital feedings:			
Fortified breastmilk (%)	74.3	43.6	90.9
Non-Fortified breastmilk (%)	2.8	5.1	3.0
Preterm formula (%)	12.0	23.1	6.1
Term formula (%)	1.7	23.0	0
D. Post D/C* predominant feedings:			
Non-Fortified breastmilk (%)	16.3	12.5	6.1
Breastmilk & post D/C* formula (%)	29.4	0	42.4
Breastmilk & term formula (%)	14.5	0	12.1
Post D/C* formula (%)	24.9	67.2	36.4
Term formula (%)	14.3	17.2	3.0

* post D/C = post discharge.

**Figure 1:**
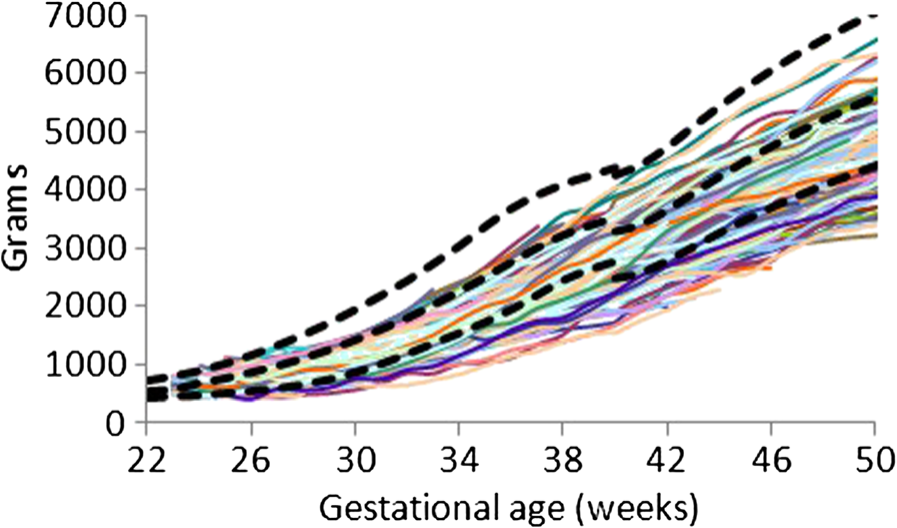
Weight gain patterns of the 23–25 week Prem Growth study infants with the Fetal-Infant Growth Reference 2013 (bold curves, 3rd, 50th & 97th percentiles), which was based on a 6 country meta-analysis of intrauterine growth (22 to 40 weeks).

**Figure 2:**
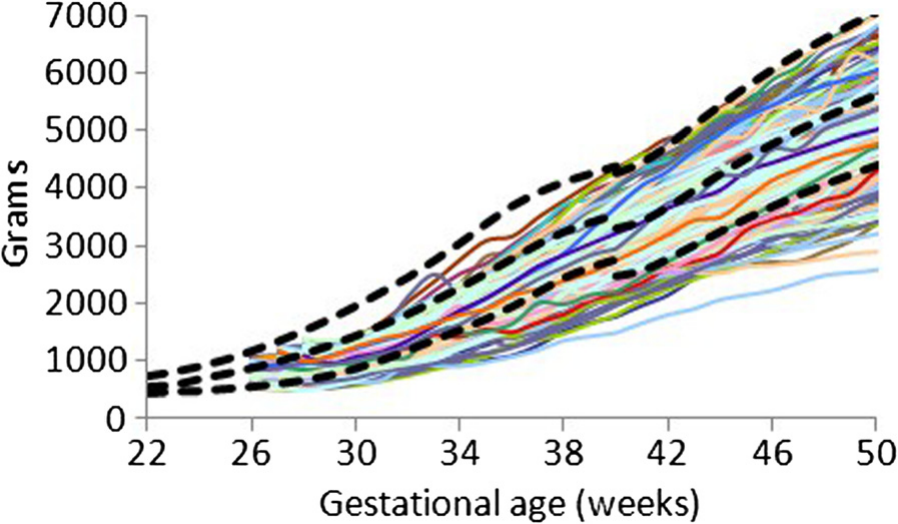
Weight gain patterns of the 26–28 week Prem Growth study infants with the Fetal-Infant Growth Reference 2013 (bold curves, 3rd, 50th & 97th percentiles), which was based on a 6 country meta-analysis of intrauterine growth (22 to 40 weeks).

**Figure 3:**
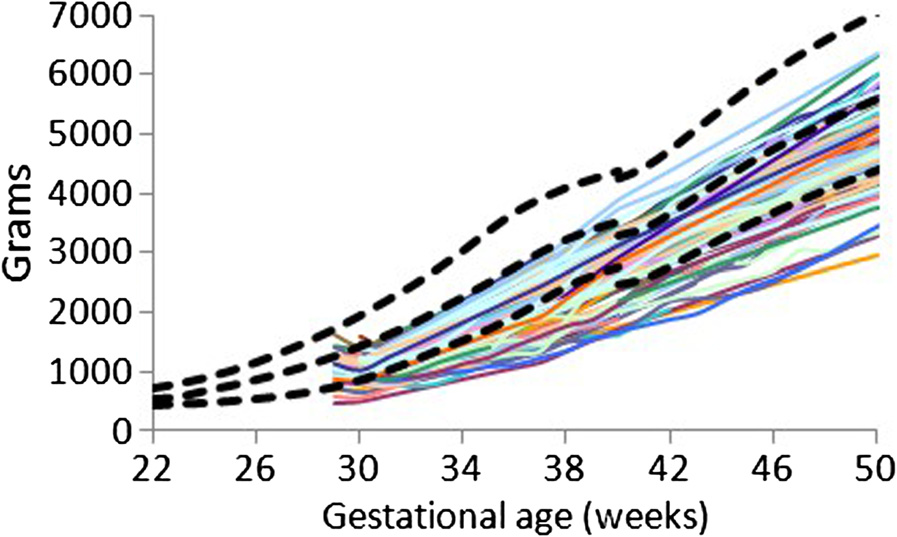
Weight gain patterns of the 29–31 week Prem Growth study infants with the Fetal-Infant Growth Reference 2013 (bold curves, 3rd, 50th & 97th percentiles), which was based on a 6 country meta-analysis of intrauterine growth (22 to 40 weeks).

The individual (Figures 1, 2, and 3) weight gain trajectories of the preterm infants plotted together with the FIGR reveal that growth in some infants remained below the 3rd percentile, while some infants in each cohort had weights greater than the median (50th percentile) after 40 weeks, and some in the 26–28 week cohort achieved weight gain up to the 97th percentile before 50 weeks.

**Figure 4:**
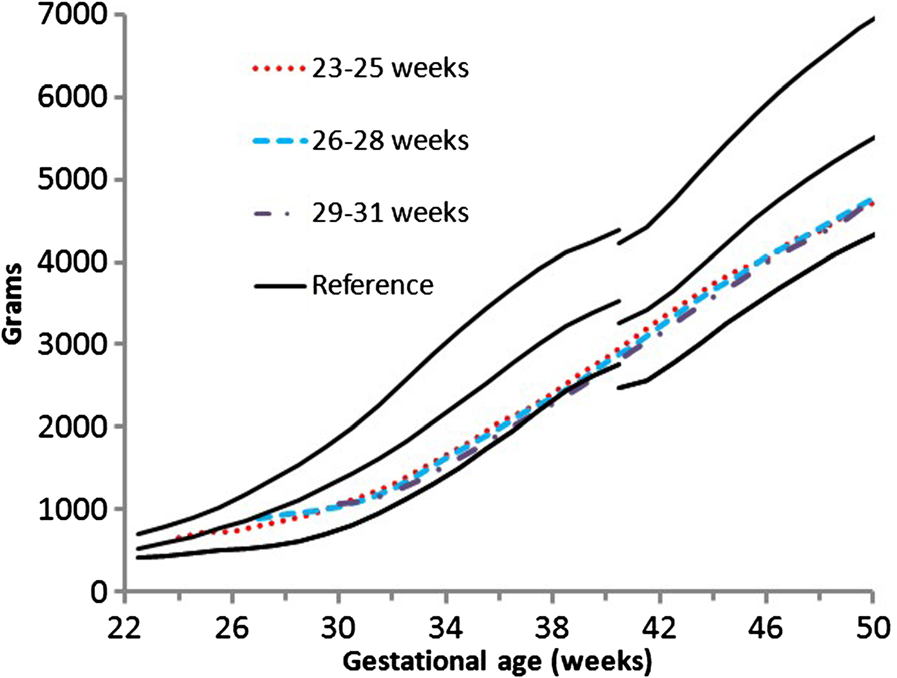
Median weight gain patterns of the 23–25 (red dot), 26–28 (blue dash), and 29–31 (purple dash dot) week Prem Growth study preterm infants with the Fetal-Infant Growth Reference 2013, which was based on a 6 country meta-analysis of intrauterine growth (22 to 40 weeks) and the World Health Organization Growth Standard (40 to 50 weeks) (3rd, 50th & 97th percentiles).

The median weight gain curves of the PreMGS cohorts localized between the 3rd and the 50th percentiles of the FIGR curves (Figure 4). During the first week after birth, the PreMGS weight gain curves plotted lower on the FIGR curves. After the PreMGS infants regained their birth weight, the median curves of the three PreMGS cohorts followed almost identical trajectories, almost maintaining the intrauterine slope. Between 37 and 40 weeks, the FIGR curves displayed a decrease in velocity that did not appear in the weight gain pattern of the PreMGS infants. After 40 weeks, the median weight gain curves of the PreMGS infants had a slightly lower slope than the WHOGS curves.

At birth, the PreMGS infants’ median weight z-scores were close to zero, with an 11% IUGR rate (Table 4). The 29–31 week cohort, however, had a median z-score less than zero (−0.9) and a higher proportion of IUGR (26%) (p < 0.0001) at birth. All three cohorts lost weight status during hospitalization, as reflected by the fall in median z-scores by 2 weeks (median z-score at birth declined from −0.1 to −1.0 at 2 weeks), with further decreases through 37 weeks (median z-score decreased to −1.7). Through the same ages, the proportions of infants considered growth restricted increased (11% to 31% to 65%) (p all < 0.0001). By 40 weeks, the PreMGS infants had improved weight status relative to the FIGR and the WHOGS. Significantly different proportions of the PreMGS infants were considered EUGR: 55% compared to the FIGR versus 35% compared to the WHOGS (p < 0.0001) at 40 weeks. By 50 weeks, the rate of EUGR had decreased to 43%, which was a significant change compared to 40 weeks (compared to the FIGR, p = 0.0001 or the WHOGS, p = 0.006) (Table 4).

**Table 4:** Weight z--scores and percent of infants weighting less than the 10th percentile*

	**All infants**	**23 to 25 wks**	**26 to 28 weeks**	**29 to 31 weeks**
**Birth vs. FIGR-2013 **[21]				
median	−0.1	0.1	0.1	−0.9
Average	−0.2	0.0	0.0	−0.9
Standard deviation	0.8	0.8	0.8	0.6
Count	978	225	541	212
% less than 10th percentile	11%	6%	7%	26%
**2 weeks postnatal age vs. FIGR-2013**				
median	−1.0	−0.8	−0.8	−1.6
Average	−1.0	−0.8	−0.8	−1.6
Standard deviation	0.6	0.6	0.6	0.6
Count	862	193	485	184
% less than 10th percentile	31%	22%	20%	68%
**37 weeks gestational age vs. FIGR-2013**				
median	−1.7	−1.9	−1.5	−2.1
Average	−1.7	−1.9	−1.6	−2.1
Standard deviation	0.9	1.0	1.0	1.0
Count	696	169	382	145
% less than 10th percentile	65%	75%	60%	78%
**40 weeks gestational age vs. FIGR-2013**				
median	−1.6	−1.8	−1.3	−1.9
Average	1.3	1.3	1.3	1.2
Standard deviation	−1.5	−1.7	−1.3	−1.8
Count	584	144	316	124
% less than 10th percentile	55%	63%	49%	62%
**40 weeks gestational age vs. WHO Growth Standard **[16]				
median	−0.9	−1.1	−0.7	−1.2
Average	1.1	1.2	1.1	1.1
Standard deviation	−0.8	−1.0	−0.6	−1.1
Count	584	144	315	124
% less than 10th percentile	35%	39%	28%	47%
**50 weeks post-menstrual age vs. WHO Growth Standard**				
median	−1.1	−1.2	−1.0	−1.2
Average	−1.2	−1.3	−1.1	−1.3
Standard deviation	1.2	1.1	1.2	1.0
Count	461	103	256	102
% less than 10th percentile	43%	48%	41%	45%

*Z-scores and assessment of growth restriction (i.e. size less than the 10th percentile) were assigned relative to the fetal-infant growth reference (FIGR)-2013 of intrauterine measures for up to 40 weeks, the World Health Organization (WHO) Growth Standard was used for 40 and 50 weeks.

**Figure 5:**
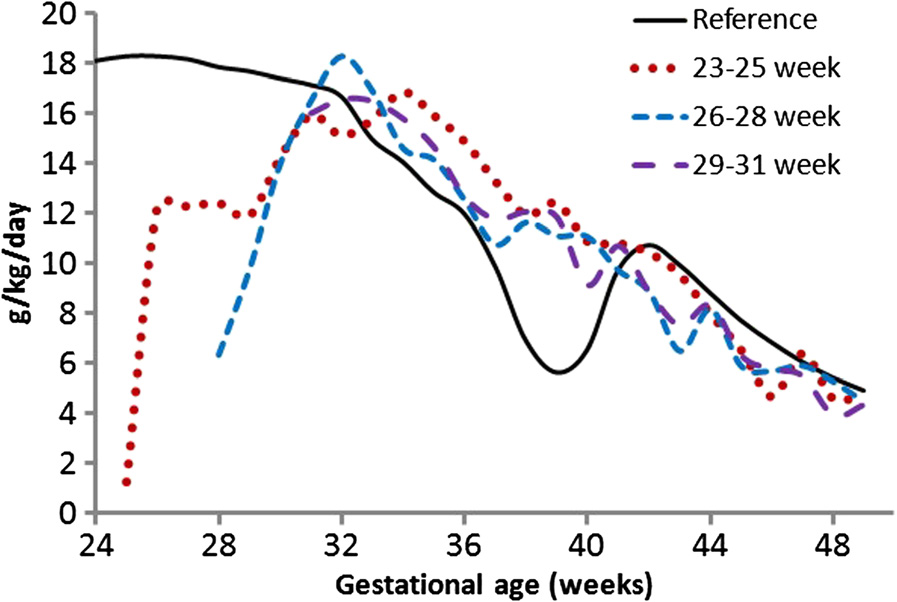
**Median weight gain velocities of the Prem Growth study preterm infants (23–25 (red dot), 26–28 (blue dash), and 29–31 (purple dash dot) week) with the Fetal-Infant Growth Reference 2013 (black), which was based on a 6 country meta-analysis of intrauterine growth (22 to 40 weeks) and the World Health Organization Growth Standard (40 to 50 weeks) (3rd, 50th & 97th percentiles), beginning at 1 week after birth.** All three cohorts weight gain velocity decreased from the higher rates at the younger gestational ages (maximum fetal reference rate = 18.3 g/kg/day at 25 weeks) to 50 weeks (infant reference rate = 4.9 g/kg/day), with a dip in the Fetal-infant Growth Reference rate around 40 weeks.

The growth velocity of the FIGR decreased with age, from high rates of 18 g/kg/day to 5 g/kg/day at 50 weeks, with one anomaly; a dip in velocity between 37 and 40 weeks (Figure 5, Table 5). Divided into two periods, the growth velocity of the median FIGR between 23 and 40 weeks was 14.8 g/kg/day, and 7.7 between 40 and 50 weeks. The dip in growth velocity between 37 and 40 weeks reached a nadir of 5.7 g/kg/day at 39 weeks before a temporary recovery to 10 g/kg/day between 40 and 43 weeks, followed by a descent to the lowest rate (5 g/kg/day) at 50 weeks.

**Table 5:** Comparison of mean weight gain velocity between the PreM Growth Study cohorts and fetal-infant estimate

**Cohort**	**mean**	**SD**	**n**	**Fetal infant estimate**	**Difference**	***P *value**
Birth to 2 weeks postnatal age						
23-25 weeks	3.8	6.6	193	18.2	−14.4	<0.0001
26-28 weeks	2.5	5.0	429	18.0	−15.5	<0.0001
29-31 weeks	6.8	4.8	160	17.3	−10.5	<0.0001
Gain 2 weeks after birth to 37 weeks						
23-25 weeks	13.3	1.7	168	16.1	−2.8	<0.0001
26-28 weeks	14.3	2.2	381	15.4	−1.1	<0.0001
29-31 weeks	14.9	2.8	144	13.5	1.4	0.46
Gain 37 to 40* weeks (40 weeks based on the FIGR-2013)						
23-25 weeks	11.5	3.4	140	7.5	4.0	<0.0001
26-28 weeks	11.4	3.1	314	7.5	3.9	<0.0001
29-31 weeks	12.3	3.8	124	7.5	4.8	<0.0001
40 to 50 weeks (40 weeks based on the FIGR-2013)						
23-25 weeks	6.9	1.7	101	6.5	0.4	0.016
26-28 weeks	7.0	1.6	256	6.5	0.5	<0.0001
29-31 weeks	6.7	1.3	100	6.5	0.2	0.09
40 to 50 weeks (40 weeks based on the WHO Growth Standard)						
23-25 weeks	6.9	1.5	101	7.4	−0.5	<0.0001
26-28 weeks	7.0	1.4	256	7.4	−0.4	<0.0001
29-31 weeks	6.7	1.2	100	7.4	−0.7	<0.0001

The growth velocity of the PreMGS cohorts matched what was seen in the FIGR, with a decrease from peak rates of 17–18 g/kg/day between 31–34 weeks down to rates of 4–5 g/kg/day by 50 weeks. There were two differences in growth velocities between the PreMGS infants and the FIGR. These differences occurred immediately after birth, and between 37 and 40 weeks when the PreMGS cohorts did not drop decrease their weight gain velocity as much as the FIGR (Figure 5, Table 5).

**Figure 6:**
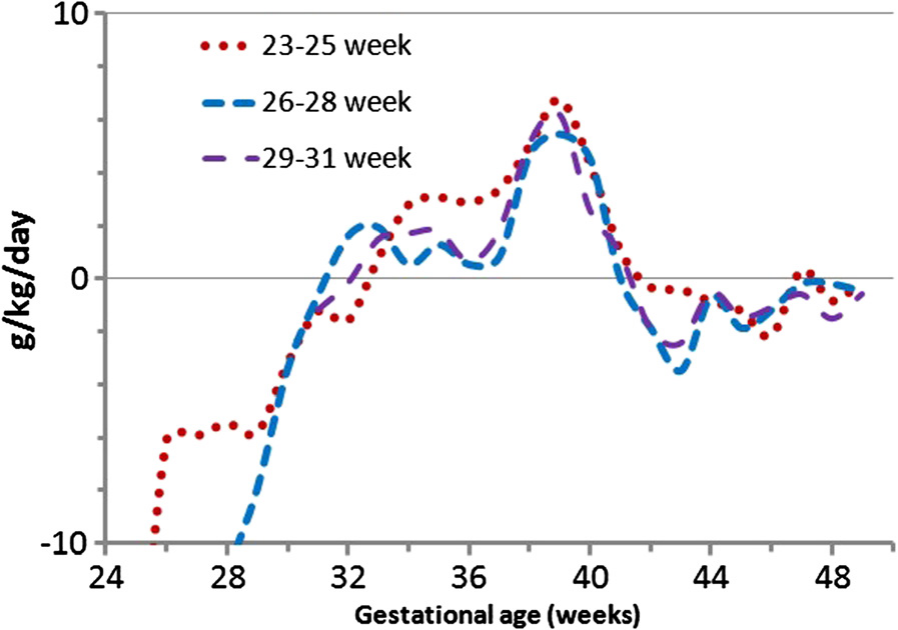
Differences in weight gain velocity between between median Fetal-Infant Growth Reference 2013 (black) (based on a 6 country meta-analysis of intrauterine growth (22 to 40 weeks) and the World Health Organization Growth Standard (40 to 50 weeks)), and the Prem Growth study preterm infants (23–25 (red dot), 26–28 (blue dash), and 29–31 (purple dash dot) week), in g/kg/day, beginning at 1 week after birth. Positive and negative values on the graph represent when preterm infant weight gain velocity was higher than or lower than Fetal-Infant Growth Reference rates, respectively.

Examining the differences between the weight gain velocity of the PreMGS infants and the FIGR revealed that all three cohorts had higher weight gain velocity than the FIGR estimate at times (Figure 6). The greatest differences between the cohorts’ weight gain and the FIGR were between 37 and 40 weeks, when all three cohorts weight gain was greater than the FIGR.

All three cohorts had lower weight gain velocities than the FIGR estimate between birth and 2 weeks, lower weight gain velocities to 37 (except for the 29–31 week cohort) (Table 5). The largest magnitude of differences in weight gain velocity between the PreMGS infants and the FIGR was between 37 to 40 weeks when the growth of the PreMGS infants exceeded the FIGR estimates by 3.9 to 4.8 g/kg/day (Table 5). Between 40 and 50 weeks, the PreMGS infants gained weight slightly better than the estimated velocity from the FIGR 40-week estimate and slightly inferior to WHOGS 40-week estimate (p < 0.02).

**Figure 7:**
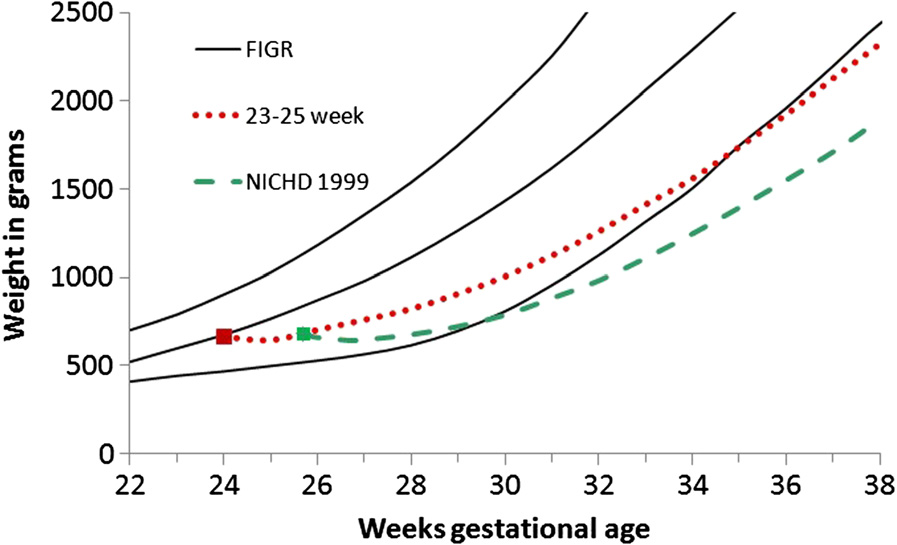
**Weight gain patterns of the National Institute of Child Health and Human Development Neonatal Research Network (NICHD) weighted average 550 and 750 gram cohorts (green dash) **[4]**and this study’s 23–25 week infants (red dot), with the Fetal-Infant Growth Reference 2013 (3rd, 50th & 97th percentiles), which was based on a 6 country meta-analysis of intrauterine growth (22 to 40 weeks).**

**Figure 8:**
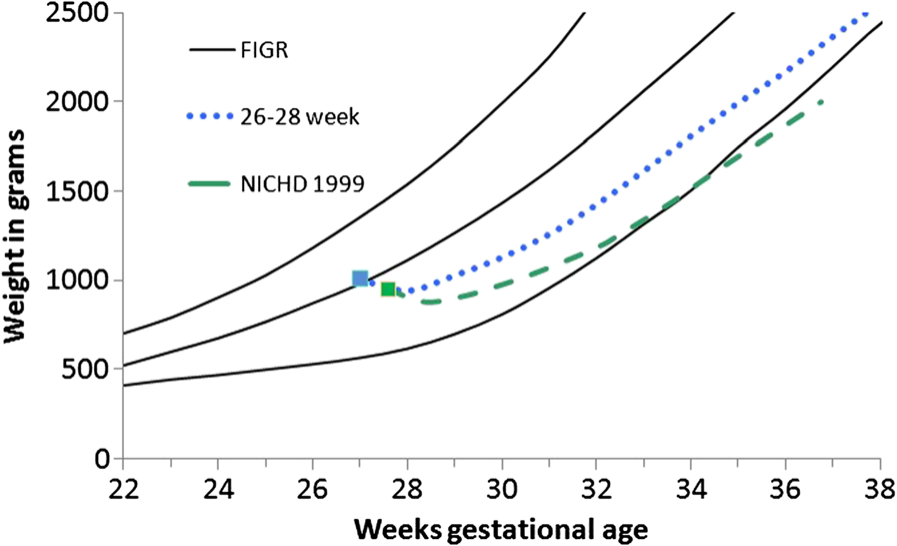
**Weight gain patterns of the National Institute of Child Health and Human Development Neonatal Research Network (NICHD) 950 gram cohort (green dash)**[4]**and this study’s 26–28 week infants (blue dash) with the Fetal-infant Growth Reference 2013 (3rd, 50th & 97th percentiles), which was based on a 6 country meta-analysis of intrauterine growth (22 to 40 weeks).**

**Figure 9:**
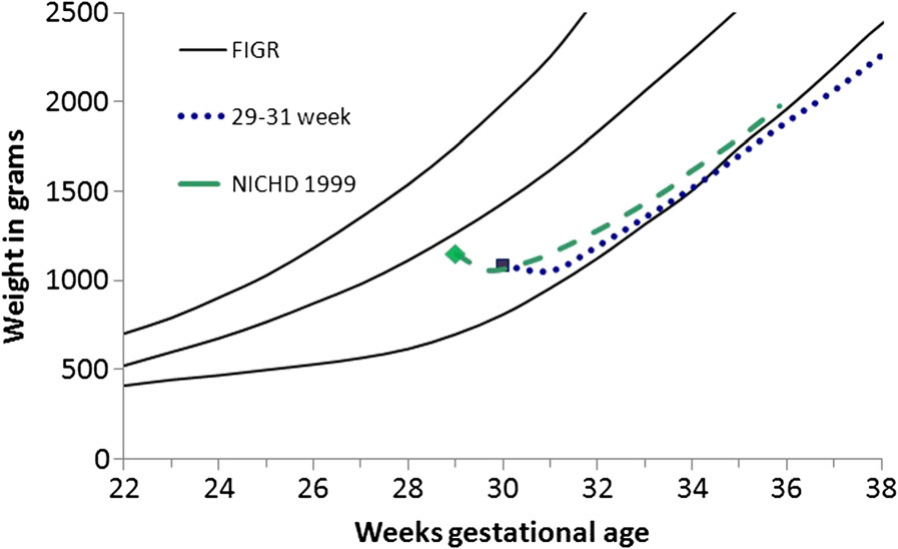
**Weight gain patterns of the National Institute of Child Health and Human Development Neonatal Research Network (NICHD) 1150 gram cohort (green dash) **[4]**and this study’s 29–31 week infants (purple dash) with the Fetal-infant Growth Reference 2013 (3rd, 50th & 97th percentiles), which was based on a 6 country meta-analysis of intrauterine growth (22 to 40 weeks).**

The comparison of the average weight gain trajectories among the PreMGS infants with the NICHD study averages revealed slightly different patterns (Figures 7, 8, and 9, Table 6). Both studies had two of the 3 cohorts mean weights below the 3rd percentile at 36 weeks (Figures 7, 8, and 9). The weight gain velocity of the PreMGS infants was greater than in two of the NICHD cohorts (Table 6). All three of the NICHD cohorts regained birthweight at older ages (mean = 13 to 17 days) compared to the PreMGS cohorts (10 to 12 days) (p < 0.0001).

**Table 6:** Mean weight gain from 1 week after birth to 34 weeks of 4 NICHD cohorts and the 3 PreM Growth Study cohorts

**NICHD cohorts**	688 g*	**950 g**	**1150 g**
N	197	191	168
Gestational age (mean)	25.4	27.6	29.0
Small for gestational age (%)	45.4	18.5	22.8
Weight gain (g/kg/d)	14.0	14.6	16.0
Regain birthweight (days)	16.6 +/− 12.3***	14.4 +/− 9.7***	13.2 +/− 8.5***
PreM Growth study cohorts	664 g	992 g	1084 g
N	193	429	160
Gestational age (mean)	24.4	27.0	29.6
Small for gestational age (%)	1.3	8.0	26.2
Weight gain (g/kg/d) **	13.1	15.2	14.8
Regain birthweight (days)	12.2 +/− 5.9***	12.4 +/− 5.1***	10.1 +/− 4.1***

* Weighted average of the NICHD 550 and 750 gram groups.

** Weight gain was calculated from when birthweight was regained to 2 kilograms.

*** p < 0.001.

## Discussion

There were important similarities and differences between the weight gain trajectories of the PreMGS infants and the reference curves. Our data revealed a close fit between the weight gain velocity of PreMGS infants and the FIGR. The weight gain velocities of PreMGS infants and the FIGR both declined steadily from the early gestation until post-term (Figure 5). The weight gain velocity of the PreMGS infants differed from the FIGR around term (Figures 5 and 6), as the infants did not follow the weight gain deceleration of the FIGR between 36 and 40 weeks. In contrast to the FIGR, the PreMGS infants continued on a relatively straight weight velocity pattern through this period. Thus, for fetal-infant growth chart development, this analysis supports the use of smoothing through the disjuncture of this period [19].

The FIGR is likely the best estimate of fetal growth until the INTERGROWTH study produces their estimates of a growth standard for preterm infants [24]. A limitation of the FIGR is that the data were cross-sectional prior to 40 weeks. Thus the estimates of growth velocity from the FIGR are based on estimates between different individuals.

Growth assessments at the time of discharge from a neonatal unit likely identifies a low point in infant growth relative to fetal references (65% of our cohort was growth restricted at 36 weeks), which did not represent how infants would be assessed a few weeks later (43% growth restricted at 50 weeks). Several research groups have previously reported poor weight gain of preterm infants relative to fetal references at 36 weeks [10-16]. Whereas other research groups have reported superior growth of preterm infants between 36 and 40 weeks relative to fetal growth references [11,12,15]. The results of this study confirmed that preterm infants can maintain weight gain velocity at higher rates than fetal growth during the period in which fetal growth slows, between 37 and 40 weeks.

Feeding preterm infants can be a challenge, and it may be important for future feeding success to have the appropriate weight gain goals to avoid overfeeding or power struggles about feeding. Authors have recommended weight gain velocity rates of 10 to 15 g/kg/day [25] or 16 to 17 g/kg/day [26]. Weight gain velocity of the fetus and the term infant are not constant, but generally decrease from highs of about 18 g/kg/day prior to 30 weeks, to about 5 g/kg/day at 50 weeks post menstrual age (Figure 5) [4-9,20,21]. Our findings confirm, a weight gain of 15 g/kg/day or more is reasonable for very low birth weight infants between birth and 36 weeks, which is approximately the age of NICU hospitalization for many very low birth weight infants. However, after 36 weeks, the weight gain velocity of the fetus falls below 15 g/kg/day, and by 44 weeks, term infant weight gain rates are below 10 g/kg/day (Figure 5).

When comparing the NICHD infants with the PreM Growth study, our conclusions must be limited because of differences between the studies in the proportions of infants who were small for gestational age and the use of different references to determine appropriateness of size for gestational age. The shorter time for the PreMGS infants to regain their birth weights compared to the NICHD infants may represent secular improvements over time, improvements in nutritional and medical care, or perhaps earlier start of parenteral nutrition as has been noted by others [27]. However, the timing of introduction of minimal enteral feedings of the infants in the PreMGS were not consistent with recommended practices to begin these trophic feeds within two days of life [28]. These PreMGS infants were not selected for superior nutrition care or weight gain, and therefore better nutrition care and growth may be achievable.

The differential growth patterns seen between the FIGR and the PreMGS infants were as expected, based on physiology. Most infants lose some weight after birth, and this weight loss is considered physiological, due to loss of extracellular water upon leaving the water-based intrauterine environment. The slowing of fetal growth velocity in late pregnancy, seen as the slight flattening of intrauterine growth curves, is thought due to limitations of intrauterine growth or placental nutrient supply, or an error due to the cross-sectional nature of the data.

There are several limitations to this study. First, we do not suggest that the growth of the infants from these three centres represent either ideal growth or nutrition support. Ideal growth for infants born prematurely has not been defined, and may be difficult to define since nutritional intakes, and thus growth rates, are defined by staff and not by demand feeding. The nutrition support received by these babies was not equivalent to Ziegler’s recommendations, that is: parenteral nutrition at birth and minimal enteral feeding begun on day 1 or 2 [28]. It might be expected that better growth than this cohort achieved would be possible on better nutrition support.

Another limitation is that it is not known whether those lost to follow-up differed from those who remained in the study. The strength of this study includes the use of recent data from infants in three cities in two countries, and the FIGR being based on a systematic review with a strict inclusion criteria [19] and data from six developed countries [4-8,21]. Third, the infants in the oldest gestational age category had a high rate of small size for gestational age and a low rate of being large for gestational age (Table 2), likely since the entrance criteria for post-discharge follow-up in Calgary favored infants less than 1250 grams at birth.

## Conclusion

Weight gain trajectories of preterm infants differ from fetal-infant estimates, in part because preterm infants do not experience the growth fluctuations of the term infant around term, particularly the growth deceleration of the fetus just prior to 40 weeks. Preterm infants born early in this century appear to follow superior weight gain patterns compared to similar sized infants who were born approximately a decade prior.

## Competing interests

The authors declared that they have no competing interests.

## Authors’ contributions

RN suggested the study, RN & TRF designed the study with assistance from all the other authors, TRF performed the statistical analysis, with assistance from ME, and wrote the manuscript. All of the authors contributed to interpret the findings and writing the manuscript, and read and approved the final manuscript.

## Pre-publication history

The pre-publication history for this paper can be accessed here:

http://www.biomedcentral.com/1471-2431/13/92/prepub
